# The influence of the environment and lifestyle on myopia

**DOI:** 10.1186/s40101-024-00354-7

**Published:** 2024-01-31

**Authors:** Sayantan Biswas, Antonio El Kareh, Mariyem Qureshi, Deborah Mei Xuan Lee, Chen-Hsin Sun, Janice S.H. Lam, Seang-Mei Saw, Raymond P. Najjar

**Affiliations:** 1 School of Optometry, College of Health and Life Sciences, Aston University, Birmingham, UK; 2 Faculty of Medical Sciences, Lebanese University, Hadath, Lebanon; 3 National University Hospital, Singapore, Singapore; 4 Department of Ophthalmology, Yong Loo Lin School of Medicine, National University of Singapore, Singapore, Singapore; 5 Singapore Eye Research Institute, Singapore, Singapore; 6 Ophthalmology and Visual Science Academic Clinical Program, Duke-NUS Medical School, Singapore, Singapore; 7 Saw Swee Hock School of Public Health, National University of Singapore, Singapore, Singapore; 8 Department of Biomedical Engineering, College of Design and Engineering, National University of Singapore, Singapore, Singapore

**Keywords:** Myopia, Epidemiology, Emmetropization, Genetics, Environment, Light, Outdoor time, Etiology, Risk factors, Progression

## Abstract

**Background:**

Myopia, commonly known as near-sightedness, has emerged as a global epidemic, impacting almost one in three individuals across the world. The increasing prevalence of myopia during early childhood has heightened the risk of developing high myopia and related sight-threatening eye conditions in adulthood. This surge in myopia rates, occurring within a relatively stable genetic framework, underscores the profound influence of environmental and lifestyle factors on this condition. In this comprehensive narrative review, we shed light on both established and potential environmental and lifestyle contributors that affect the development and progression of myopia.

**Main body:**

Epidemiological and interventional research has consistently revealed a compelling connection between increased outdoor time and a decreased risk of myopia in children. This protective effect may primarily be attributed to exposure to the characteristics of natural light (i.e., sunlight) and the release of retinal dopamine. Conversely, irrespective of outdoor time, excessive engagement in near work can further worsen the onset of myopia. While the exact mechanisms behind this exacerbation are not fully comprehended, it appears to involve shifts in relative peripheral refraction, the overstimulation of accommodation, or a complex interplay of these factors, leading to issues like retinal image defocus, blur, and chromatic aberration. Other potential factors like the spatial frequency of the visual environment, circadian rhythm, sleep, nutrition, smoking, socio-economic status, and education have debatable independent influences on myopia development.

**Conclusion:**

The environment exerts a significant influence on the development and progression of myopia. Improving the modifiable key environmental predictors like time spent outdoors and engagement in near work can prevent or slow the progression of myopia. The intricate connections between lifestyle and environmental factors often obscure research findings, making it challenging to disentangle their individual effects. This complexity underscores the necessity for prospective studies that employ objective assessments, such as quantifying light exposure and near work, among others. These studies are crucial for gaining a more comprehensive understanding of how various environmental factors can be modified to prevent or slow the progression of myopia.

## What is myopia?

**Fig. 1:**
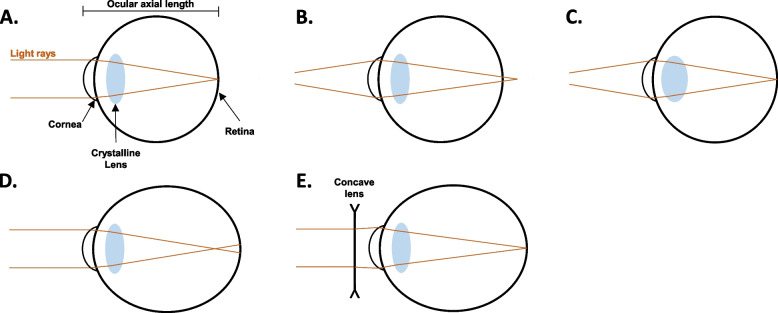
Schematic of emmetropia and axial myopia. **A** In an emmetropic eye, parallel rays of a distant object are focused on the retina. **B** When an eye is tasked to focus on a near object, without accomodation, the image of the object is focused behind the retina. **C** Accommodation can bring forward the image to focus on the retina. **D** In axial myopia, the eye's axial length has grown longer than the dioptric focus of the eye. Light rays are therefore focused in front of the retina resulting in the blurred vision of a distant object. **E** Myopia can be optically corrected using a concave lens (spectacles or contact lenses) which diverges the light rays and moves the image into focus on the retina

Myopia or near-sightedness is a refractive error that is predominantly caused by a mismatch between the optical power of ocular components (i.e., the cornea and the crystalline lens) and the axial length (AL) of the eye whereby light entering the eye is focused anterior to (in front of) the retina, leading to the blurred vision of distant images [1, 2]. In axial myopia, an excessive antero-posterior elongation of the eyeball occurs with thinning of the retina, choroid, and sclera [1] (Fig. 1). This excessive axial elongation is hypothesized to trigger sub-foveal chorio-retinal stretching, increasing the risk of sight-threatening ocular diseases such as posterior staphyloma, retinal degeneration, and glaucoma [3]. On the other hand, refractive myopia is predominantly associated with steepening of the cornea and lens curvature which increases the optical power of the eye [1].

Blurred distance vision due to myopia can be corrected using negative (concave) spectacles or contact lenses that refocus the image on the retina [4]. The power of the corrective lens in diopters (D) reflects the degree/severity of myopia [5]. For an eye to be considered myopic, the spherical equivalent refractive error (spherical refraction + 0.5 * cylindrical refraction) with ocular accommodation relaxed must be ≤ −0.50 D. In high myopia, the spherical equivalent refractive error when ocular accommodation is relaxed is ≤ −5.00 D [6].

## Myopia is a public health concern

The prevalence of myopia is not homogeneous across the globe. In school children (6–19 years old), the highest myopia prevalence was reported in Asia (60%; including East Asia (73%)), followed by North America (42%), Europe (40%), South America (~10%), and Africa (3.4–4.0%) [7]. In young adults, the prevalence is much higher in urban East Asian countries (81.6–96.5%) than in the rest of the world (12.8–35.0%) [8]. In comparison, the prevalence of adult myopia was 19.4–41.8% among East Asians, 17.2–36.5% in the rest of Asian countries, and 11.4–35.1% among non-Asians [8]. The worldwide prevalence of myopia is on the rise for reasons that are still not well understood [7, 9–11]. A systematic review and meta-analysis of 145 studies worldwide on myopia prevalence predicted that by 2050, half of the world population (4,758 million people) will be myopic and ~10% of the world population (938 million people) will have high myopia. [12]. In Europe, however, findings are mixed, with both reports of an increase [13] and no change in myopia prevalence [14].

In addition to being a public health concern, myopia is also a health economic burden. There are several estimates for the global financial burden related to myopia (i.e., the health expenditure and loss of productivity), all of which are in the range of several hundred billion dollars per year [9]. High myopia increases the risk for other sight-threatening ocular conditions like retinal detachment, glaucoma, and cataract [15, 16]. Also, both uncorrected myopia and pathologic myopia (characterized by lesions in the fundus like staphyloma, neuropathy, and maculopathy) are associated with reduced quality of life [9]. Hence, investigating the disease process, epidemiology, etiology, and risk factors for myopia in addition to emerging therapeutic strategies for this condition is essential in halting the myopia epidemic.

## Clinical features of myopia

Refractive error is typically measured by means of objective (i.e., autorefractor) or semi-objective (retinoscopy; objective from the patient’s perspective but not the operator’s as it requires examiner skill) techniques as a starting point before subjective refraction. Accommodation induced by the eye’s ciliary muscles can influence refractive error, inflating the prevalence and degree of myopia by 0.63 to 0.89 D in children with active accommodation [17]. The gold standard for accurate subjective refraction is cycloplegic refraction, which involves using pharmacologic agents that temporarily paralyze the ciliary muscles and accommodation. Alternatively, open-field autorefractors reduce the effect of residual accommodation and instrument myopia, are more repeatable and precise, and minimize investigator bias [17].

AL is measured as the axial distance between the anterior corneal surface to the retina (inner limiting membrane or retinal pigment epithelium depending on the technique used) along the line of sight. AL is highly correlated with the refractive error [18] with approximately a 2.3 D increase in myopia associated with a 1-mm increase in AL and vice versa [19]. AL can be measured using ultrasound biometry, optical biometry, and optical coherence tomography (OCT) techniques [17].

An increase in the corneal steepness and power (i.e., decrease in radius of curvature) increases myopic refraction and vice versa [20]. Corneal curvature and the corresponding power can be measured using keratometry and a wide range of corneal topography and anterior segment imaging devices.

Choroidal (inner sclera to outer retinal pigment epithelium (RPE)), retinal (internal limiting membrane to Bruch’s membrane), and sub-foveal scleral (chorio-scleral interface to the outer scleral border) thinning are also observed in myopic eyes. These structural assessments can be measured using posterior segment OCT devices with enhanced depth imaging (EDI) [21], wide field/high-penetration swept source OCTs [22, 23], and magnetic resonance imaging [24].

## Emmetropization and myopia development

Emmetropization is a visually guided phenomenon that occurs from birth and regulates axial ocular growth to match the eye’s focal length with its focal power. Abnormal emmetropization, or its maintenance, is the fundamental problem in myopia development. This can be induced experimentally in a variety of ways. Depriving the eye in animal models from spatial vision results in ocular axial elongation and subsequent form deprivation myopia (FDM). Form deprivation can be induced in animals by eyelid sutures [25–28], translucent diffusers, or frosted goggles which reduce the sharpness and contrast of retinal images [29]. Clinically, FDM is reported in ocular conditions such as congenital ptosis and cataracts, which deprive the eye of visual stimulation [30–32]. Compensatory ocular growth towards emmetropization is also driven by hyperopic or myopic defocus at the retina. Several animal species, most commonly chicks wearing positive or negative lenses were found to exhibit a rapid change in eye growth to compensate for the defocus and attain emmetropia [33]. Hyperopic defocus (image behind the retina) is generated by negative lenses which stimulate axial elongation, while myopic defocus (image in front of the retina) generated by positive lenses inhibits axial elongation [34–36]. The eye can decipher between myopic and hyperopic blur/defocus and alter its growth accordingly.

Emmetropization has two phases, a rapid infantile phase, and a slower juvenile phase. The **rapid infantile phase** starts between 3 and 9 months of life, where a myopic shift in refraction towards low hyperopia or emmetropia occurs [37, 38]. During that phase, a fast increase in AL (~5 mm) is accompanied by compensating changes in ocular structures resulting in corneal and lens power reduction [39]. Concomitantly, new-born (3 months old) have an average cycloplegic hyperopic refraction of about +2 D, which rapidly reduces to +0.75 D by the time they reach 3.5 years of age [40]. The AL increases from an average of 15 mm in new-born to 24 mm by early adulthood and is counteracted by an equal and opposite change in corneal and lens power [41]. The **slower juvenile emmetropization phase** starts at 3 years and continues till adolescence [42]. Similarly, during this phase, AL grows along with changes in the cornea and lens, albeit at a much slower rate [42]. The majority of myopia onset occurs during this phase, often between ages 6 and 9 years [43, 44], followed by a rapid phase of myopic shift in refractive error, which plateaus by early adulthood [40]. Myopia onset, after the primary emmetropization period, can result from the failure to maintain an emmetropic state and not a failure of the emmetropization process [40]. The earlier the age of myopia onset, the higher the risk for high myopia and associated sight-threatening conditions. Hence, delaying the onset of myopia may delay or prevent pathological myopia [45].

## Genetic factors influencing myopia

Parental myopia significantly influences a child’s likelihood of developing myopia. For instance, the proportion of children developing myopia is 32.9%, 18.2%, and 6.3% with two, one, and no myopic parents, respectively [46]. Among a predominantly white population (89.1%), the odds of being a myope also increase from one (odds ratio (OR), 3.32) to two parents (OR, 6.40) with myopia [46]. Whereas in a mixed population, the ORs were 1.42 for one parent, 2.70 for two parents, and 3.39 for two parents with early onset myopia, respectively [47]. These findings are corroborated by longitudinal cohort studies with follow-ups of 7 and 22 years [48, 49]. In addition, genome-wide association studies (GWAS) and their meta-analyses have helped identify several single nucleotide polymorphisms (SNPs) associated with myopia [50–53]. Nonetheless, common SNPs identified through GWAS thus far can only explain 18.4% of spherical equivalent heritability [51]. The effect sizes of SNPs associated with myopia are small, in the order of ±0.1 D [54]. Thus, association with parental myopia (i.e., inheritance or heritability) does not necessarily mean genetics are causative of myopia. The surge in myopia prevalence worldwide, occurring without significant genetic changes between generations, suggests a considerable role of behavior-influenced environmental and lifestyle factors in myopia development [55, 56]. In the following paragraphs, we highlight some of the most prominent environmental and behavioral factors that have emerged as significant contributors to the onset and progression of myopia in children.

## Environmental factors influencing myopia

### Time spent outdoors

Both cross-sectional and longitudinal studies have reported a significant association between increased time spent outdoors and reduced myopia prevalence. Numerous cross-sectional studies including the Sydney myopia study (SMS) (*n*=2367, 12 years old) [57], the Singapore Cohort of Risk factors for Myopia (SCORM) (*n*=1249, 11–20 years old) [58], the Beijing Myopia Progression Study (BMPS) (*n*=386, 6–17 years old) [59], among others, have independently reported a significant association between increased time outdoors and lower myopia rates and vice versa. Likewise, longitudinal studies including the Avon Longitudinal Study of Parents and Children (ALSPAC) (*n*=4837–7737, 7–15 years old) [60], the Sydney Adolescent Vascular and Eye Study (SAVES) (*n*=2103, 6 and 12 years old) [61], the Collaborative Longitudinal Evaluation of Ethnicity and Race (CLEERE) (*n*=731, 6–14 years old) [62], and the Orinda Longitudinal Study of Myopia (OLSM) (*n*=514, 8–9 years old) [63], confirmed these associations between delayed myopia onset and increased time spent outdoors. Australian children spending more time on outdoor activities (13.75 vs 3.05 h/week) [64] and longer daily outdoor light exposure (105 vs 61 min/day) [65] were found to have a lower prevalence of myopia (3.3%) [64] than Singaporean children (29.1%). Interventional randomized controlled trials (RCT), two in Chinese (*n*=6925, 6–9 years old, *n*=3051, 6–14 years old, and *n*=1903, 6–7 years old) [66, 67] and two in Taiwanese (*n*=571, 7–11 years old and *n*=693, 6–7 years old) [68, 69] school children demonstrated that incorporating 40 to 80 min of interrupted recess time outdoors reduces myopia incidence. However, it should be noted that the reporting of light exposure using questionnaires in these studies may be more prone to reporting bias compared to objective measures [70]. A recent RCT [71] evaluating the protective effect of 0, 40, and 80 min of additional time outdoors among 6–9 years old Chinese school children for over 2 years observed a dose-response relationship between the outdoor exposure time and myopia onset and progression. The protective effect was associated with the objective measurement of both the duration of exposure and light intensity. Likewise, recent systematic reviews and meta-analysis [72–76] along with their overviews [77, 78] reinforce the compelling protective impact of time spent outdoors against myopia and highlight a 2–5% reduced OR of prevalent myopia and 24–46% reduction in relative risk of incident myopia for every additional hour of outdoor time per week.

While parents are being advised to promote outdoor activity for their children, a systematic review of evidence suggests increased time outdoors is effective in preventing the onset of myopia but is not effective in slowing the progression in eyes that are already myopic [73]. However, interventional studies have demonstrated that myopia progression can indeed be mitigated by increasing the time spent outdoors [66–69], and a recent meta-analysis has reaffirmed this effect, revealing a pooled reduction effect of 0.13 to 0.17 D in myopic refractive error per year [78]. Conversely, the substantial uptick in indoor time and a reduction in outdoor activities among schoolchildren during the COVID-19 pandemic-related home confinement was linked to a rise in both the incidence of myopia and the rate at which it progressed [79, 80]. It is worth mentioning that some studies like the CLEERE [81], Anyang Childhood Eye Study (ACES) [82], amongst others [83–85], found no associations between the duration of time spent outdoors, the incidence of myopia, and its progression. These disparities in findings may stem from variations in cohort age, study duration, and research design, as well as differences in the criteria used for classifying myopia.

The protective effect of time outdoors against myopia has primarily been attributed to the level and spectral compositions of daylight (i.e., high light levels, broad spectral distribution) [29, 86, 87], the visual-spatial characteristics (i.e., high spatial frequency), and accommodative profiles (i.e., less variation and demand) [88, 89] of the environment outdoors, all lacking in most indoor environments, especially in schools. In the sections below, we discuss the “independent” effects of these environmental features on myopia.

### Features of the lighting environment

Lighting characteristics such as intensity, spectral composition, duration, pattern, and timing can synergistically affect ocular growth and development [29].

#### Light levels

Indoor illuminances generally range between 10 and 1000 lux, whereas outdoor light levels on a cloudy day or under shade can vary between 10,000 and 30,000 lux [86] and reach more than 100,000 lux [90, 91] on a sunny day. Low daily light exposure measured using objective wrist-worn light sensors has been associated with greater axial elongation and myopia [92]. Concurrently, exposure to both long-term [93] and short-term (30–120 min) light [94, 95] of moderate levels of illumination (500–1000 lux) induces a significant reduction in axial elongation and an increase in choroidal thickness (CT) in young adults. Low illumination levels (359 vs 671 lux) in the nursery (4–5-year-old children) [96] and the lowest daylight factor in elementary (6–7-year-old children) [97] school classrooms were suspected to be associated with myopia and axial elongation. Similar results echoed in a 1-year RCT (*n*=1713, 6–14-year-old children) with higher ambient light levels of 558 lux vs 98 lux at the desk and 440 lux vs 76 lux at the blackboard being protective against the onset and progression of myopia and ocular axial elongation [98].

Animal studies further provide evidence supporting the protective effect of high illuminance against myopia. Chicks [36, 91, 99, 100], infant monkeys [101], and guinea pigs [102] exposed to high-intensity light (≥10,000 lux) for 5–6 h/day showed reduced experimental myopia development.

#### Spectral composition of light

**Fig. 2:**
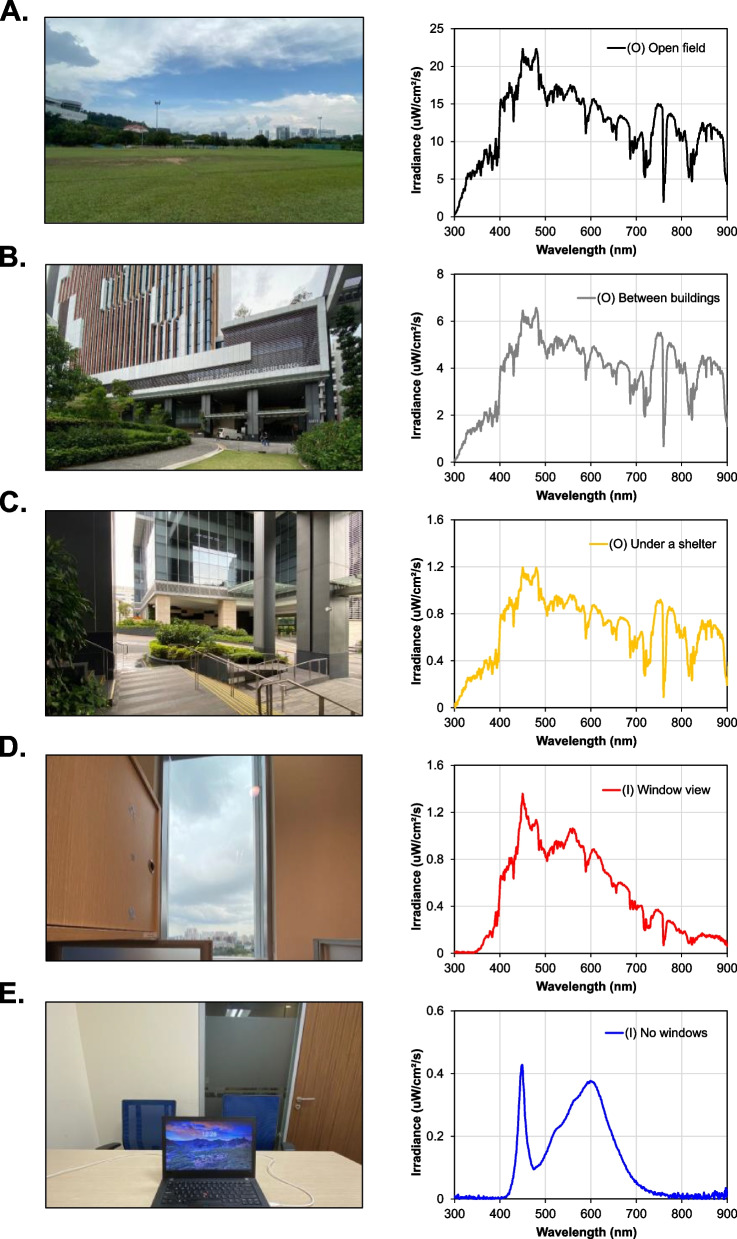
Spectral power distribution of light in different environments, both outdoors (**O**) and indoors (**I**)

**Fig. 3:**
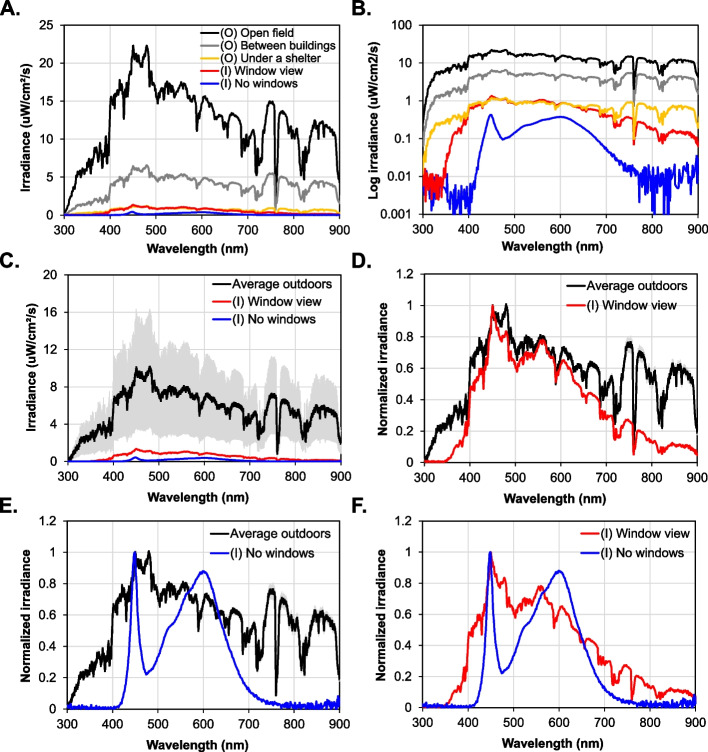
A direct comparison of the levels and spectra of light measured indoors and outdoors. **A** Light levels outdoors are significantly higher than light levels indoors. **B** The light spectrum outdoors remains fairly unaltered when measured in different locations. Conversely, light levels outdoors can decrease by ~1 log unit between an open field and a denser building area or even indoors looking out from a window. Conversely, light levels can drop by more than 10 log units in a room equipped with artificial lighting. **C** The spectral power distribution of the average measurements outdoors (±SEM) compared to indoor scenarios. **D**, **E**, and **F** Normalized spectral power distribution of light outdoors compared to light indoors. While the spectrum remains fairly similar between 400 and 650 nm, windows block a considerable amount of ultra-violet (<400 nm) and near-infrared or infrared light (>650 nm) (**D**). Similar differences are observed between traditional indoor LED lighting (CCT: 4000 K) in addition to reduced composition in wavelengths between 400–440 nm and 480–560 nm (**E**). Similar observations can be made when comparing artificial lighting to sunlight seen through a window (**F**)

In addition to light levels, the spectral composition of light can affect emmetropization and myopia development. Compared to the most frequently used artificial light sources indoors (e.g., fluorescent, light emitting diodes (correlated color remperature (CCT): 2000 K – 6500 K), halogen), the spectrum of sunlight is dynamic across the day and has a fuller distribution of wavelengths [87]. Natural sunlight contains ultra-violet (UV), near-infrared, and infrared (IR) wavelengths of light (Fig. 2A–C, Fig. 3). The effect of different wavelengths of light on myopia development is still not well understood, especially in humans. Torii et al. showed that UVA light (360–400 nm), absent in commonly used indoor lights (Fig. 2E), could suppress myopia progression and reduce axial elongation in humans through the upregulation of the transcription factor early growth response factor-1 (EGR-1), encoded by the EGR1 gene [103], and via neuropsin (OPN5) stimulation in mice [104]. In an RCT on 6–12-year-old Japanese children (2-year follow-up), violet light-transmitting glasses were found to reduce axial elongation by 21.4% compared to glasses that do not transmit violet light. However, the effect was significant only in the sub-group of those performing <180 min of near work or first-time glass users [105]. A 6-month randomized pilot study on using violet light-emitting glasses for 3h/day had a small but significant protective effect on AL elongation in 8–10-year-old children. However, the sample size was only 10 and the effect was not significant for younger (6–7 years) or older (11–12 years) children [106]. Further investigations are warranted to assess both the efficacy and safety of UVA light exposure for myopia control. Similarly, in a laboratory setting, exposure to short wavelength blue light (460 nm) for 1 h was found to reduce axial elongation in young adults (20–32 years) compared to both green (521 nm) and red light (623 nm) [107]. Likewise, the stimulation of blind spots (optic nerve head) using flickering blue light (peak 450 nm) for 1 or 10 min increases retinal activity (increases *b* wave amplitude of bipolar cells) [108]. This intervention was built around the fact that blue light would stimulate melanopsin present in the axons of intrinsically photosensitive retinal ganglion cells (ipRGCs) having synaptic connections with dopaminergic amacrine cells, thus modulating the release of the neurotransmitter dopamine (DA) [108]. With DA regulating ocular growth (see the section “potential mechanisms for light-driven myopia prevention and control” below), this effect may have an implication for myopia control. It is also possible that longitudinal chromatic aberration focusing various wavelengths of light differently relative to the retina, alters the outdoor effect on myopia by providing a visual cue (directional) during emmetropization [109]. A recent clinical study reported that 2 h of non-objective outdoor sunlight exposure (average 6000–50,000 lux) promotes choroidal thinning and retinal thickening compared to indoors (350 lux) and dark (<0.1 lux) [110]. This contrast from previous findings of a transient increase in CT on the application of light exposure (indoor LEDs) [94, 95] is probably because of the differences in experimental protocol and lack of control over the confounders such as the visuo-spatial environment [89], caffeine intake [111], smoking [112] preceding, and during the 2-h long unsupervised outdoor activity. On the other end of the visible light spectrum, several randomized clinical trials (6–24 months of follow-up) have shown great promise that repeated low red light (RLRL) (640 nm) administered for 3 min twice a day can slow myopia progression in children aged 7–15 years old [113, 114]. The strength of evidence for RLRL was, however, low, with large rebound effects reported after its discontinuation [115]. Furthermore, there have been safety concerns associated with RLRL, including reports of an isolated case of retinal damage that may potentially be linked to the procedure [116].

In addition to studies in humans, experimental studies in animal models have also shown that different wavelengths of light have distinct effects on myopia development. While shorter wavelengths of light (290–495 nm) [103, 104, 117–125] have been shown to be protective against experimental myopia in chicks, mice, and guinea pigs, longer wavelengths (570–636 nm) of light [126–131] were found to be protective in tree shrews and some non-human primates. This variation in spectral response among different species may be attributed to several factors, including differences in retinal photoreceptor structure, sensitivity, and variations in experimental protocols such as the duration of light exposure and its intensity [29, 124]. In addition, recent studies demonstrated that sun-like and blue-enriched light spectra, even at low-moderate illuminances (200–400 lux), can slow the development and accelerate recovery from FDM in chicks [132, 133].

#### A comparison of light levels and spectra between different environments

As mentioned in the paragraphs above, light levels and spectra differ greatly between indoors and outdoors [29, 134]. To better highlight these differences, we performed the study below. The spectral power distribution of ambient light was measured at the eye level in different outdoor and indoor locations using a calibrated spectroradiometer (ILT950, International Light Technologies, Peabody, MA, USA). In an open field, outdoor light intensity is at its highest, with the sun serving as the sole light source (Fig. 2A). As urbanization gradually becomes evident, such as between buildings with open tops (Fig. 2B), the spectral distribution remains similar but with a diminished light intensity. Further urbanization, as seen when measuring light outdoors but under a shelter with a covered roof (Fig. 2C), results in reduced ambient light intensity while preserving a similar spectral distribution. However, the introduction of modern urbanized buildings, even with a window view, leads to a drastic alteration in the indoor spectral distribution of available light, although light intensity remains comparable (Fig. 2D). Subsequently, when we bring the full extent of urbanization indoors in a setting devoid of windows and illuminated by LED light (Fig. 2E), we observe a further modification characterized by both decreased intensity and a shift in the spectral distribution, now manifesting as two peaks at approximately 450 nm and 600 nm.

When performing a direct comparison of light levels and spectra measured both indoors and outdoors (Fig. 3), outdoor light levels are significantly higher than those encountered indoors (Fig. 3A). The outdoor light spectrum remains relatively consistent across different outdoor locations, but light levels can decrease by at least one log unit when transitioning from an open field to a more densely built area or even when viewing from indoors through a window (Fig. 3B). Conversely, indoor light levels can drop by over 10 log units in spaces with artificial lighting (Fig. 3B, C). The natural light spectrum outdoors and indoors (through a window) remains fairly consistent between 400 and 650 nm, while windows block a significant portion of ultraviolet light (<400 nm) and near-infrared or infrared light (>650 nm) (Fig. 3D). Similar differences are observed when comparing traditional indoor LED lighting (CCT: 4000 K), with reduced composition in wavelengths between 400–440 nm and 480–560 nm (Fig. 3E). Comparable distinctions can be observed when contrasting artificial lighting with sunlight seen through a window (Fig. 3F).

Given the evident disparities between natural outdoor light and current indoor lighting, it has become imperative to urgently curate and tailor indoor lighting environments, whether artificial or natural, to promote healthier ocular development in children [135]. This is critical within educational settings, such as classrooms, where children predominantly spend their daylight hours.

#### Timing, duration, and pattern of light exposure

Experimental animal research findings suggest that the timing, duration, and pattern of light exposure can influence myopia. For instance, moderate light during mid-day (2000 lux) is more effective at reducing myopia than evening exposure in chickens [136]. Longer light exposures (2000 lux for 10 h) can be more effective than an equivalent dose of shorter, but brighter exposures (10,000 lux for 2 h), irrespective of the exposure time-of-day (morning, mid-day, or evening) [136], whereas intermittent exposure to high illuminances of light (15,000 lux) may be more effective than continuous light of equal duration and illuminance against FDM in chickens [137]. On the other hand, constant light (i.e., for 24 h) can disrupt the emmetropization process [138] and evening exposure to light (700 lux) was reported to disrupt the circadian rhythm of ocular growth [139, 140]. The frequency of a light flicker also modulates eye growth with low frequency stimulating and high frequency reducing eye growth [141]. It is worth mentioning that the distinctive effect of these light features has been understudied in humans.

#### Potential mechanisms for light-driven myopia prevention and control

##### Modulations in ocular neurotransmitters and signaling molecules:

DA, a neuromodulator, is the most widely studied neurotransmitter and is proposed to influence eye growth and the emmetropization process [142]. DA is released by the amacrine and/or inter-plexiform cells of the retina [143, 144] and has a dose-response relationship with the intensity of light [145–147]. Even intermittent light exposure was found to be more effective than continuous light of equal duration possibly because of the activation of retinal ON and OFF pathways by flickering light, stimulating DA release [148]. Animal studies implicated DA activity to mainly mediate via the D2 receptor pathway, although D1 and D4 (D2-like receptors) also play some role in refractive development, which remains controversial [149]. Even though DA levels get directly influenced by the duration and intensity of light, DA is also found to be released under dark conditions, following the circadian pattern of its release [150] and rod cell activation [151]. Furthermore, DA agonists [152, 153] and antagonists [99] were also used to support the role of DA in axial elongation. However, there are no clinical studies linking DA and myopia due to the obvious limitation of accessing human ocular tissue. DA is also known to modulate CT and axial growth by triggering the release of other neurotransmitters, such as nitric oxide (NO) [142, 154, 155]. NO was found to be dependent on light levels and reduces FDM [156]. Other neurotransmitters and signaling molecules linked with light and myopia are atropine, 5-hydroxytryptamine (5-HT), EGR-1 (ZENK), gamma-aminobutyric acid (GABA), retinoic acid (RA), melanopsin, and ipRGCs [29, 142, 155].

##### Blood flow

DA enhances retinal perfusion and choroidal blood flow in humans [157]. Reduced ocular blood flow can be implicated as a potential cause for choroidal and retinal thinning and associated eyeball elongation in myopia. To support this theory, lower ocular blood flow has been frequently reported in myopic eyes [158]. However, it is unclear whether reduced blood flow is a primary change that causes secondary thinning of the choroid and retina or quite the opposite, i.e., the mechanical stretching of the eye reduces its wall thickness and causes a secondary lower demand for oxygen.

##### Vitamin D

Vitamin D is available in small amounts from food such as fish and eggs, but the majority is synthesized in the skin on exposure to sunlight (UVB). Several cross-sectional studies found lower levels of vitamin D in myopes compared to non-myopes [159–163]; however, subsequent studies found no such evidence [164, 165]. A review of time outdoors, vitamin D, and its association with myopia found an interrelation but without any biological plausibility [166]. Moreover, myopia is not a characteristic feature associated with rickets (vitamin D deficiency) which suggests vitamin D may indicate time outdoor levels (UVB exposure) but not have any protective effect itself [167].

### Spatial frequency and other environmental visual features

The spatial frequency of the visual environment strongly differs between indoor and outdoor sceneries [88]. Urban outdoor environments were found to lack greenery and high spatial frequency with defocused retinal images which is similar to the image generated using diffusers to induce FDM in animals [89]. On the contrary, images of greenery contain significantly higher spatial frequency content [89]. The level of residential greenness in Spain and China has been reported to reduce spectacle use and the risk of myopia among preschool and school children [168–170]. While these benefits could potentially be attributed to a reduction in daily screen time [168], it is important to consider that changes in spatial frequency and exposure to natural light may also be contributing factors. Animal studies found intermediate and mixed spatial frequencies to reduce FDM compared to both high and low spatial frequencies in chickens [171, 172]. Besides, accommodative micro-fluctuations were also found to be dependent on the spatial frequency of images, with the lowest fluctuation at medium spatial frequency [173, 174]. Further studies are required to elucidate the impact of spatial frequency on ocular growth and myopia development in children.

Changes in color and luminance contrast are also found to provide cues for defocus and thus affect emmetropization. Higher red contrast in the defocused retinal image than the green and blue components under simulation can relax accommodation and reduce eye growth, whereas higher contrast of the blue component compared to the green and red was found to increase accommodation and promote eye growth [109].

### Near work

Among children, indoor activities primarily consist of tasks such as reading, writing, and using digital devices at close but variable distances. When these distances are converted into diopters (which is the reciprocal of the distance in meters), it becomes evident that the indoor visual environment exhibits significantly greater dioptric variations compared to outdoor settings [88]. Ocular accommodation increases directly with the proximity of viewed objects and is due to an increase in lens convexity (in addition to pupil constriction and convergence of the eyes during the accommodation reflex) with results in the increase of the optical power of the eye [175]. The accommodation demand profile for even basic tasks like reading a book or viewing a computer screen fluctuates by several diopters, even across the retina (i.e., from the central point of fixation (the fovea) to the peripheral retina) [88]. When the accommodation response is considered along with this dioptric variation, outdoor environments have more uniform retinal focus than indoors which is associated with greater levels of defocus, especially in the peripheral retina. In addition, the average mismatch between the accommodation response and demand (known as the accommodative lag or error) across indoor visual scenes can be 2.88 D for reading and 0.14 - 1.77 D for computer use (with superior and inferior retinal hyperopic defocus), while it can be around 0.05 D for outdoor tasks [88]. Briefly, accommodation is more predominant indoors compared to outdoors which is associated with long viewing distances, fewer variations in accommodative demand, and more uniform retinal focus [88].

More time spent indoors and close reading distance have been associated with a higher risk of myopia among school children [176]. Notwithstanding, it is important to highlight that the correlation observed between myopia and near-work activities in studies does not necessarily establish a cause-and-effect relationship. In fact, it is plausible that the development of myopia could potentially lead to children spending more time indoors engaging in near-work activities and less time participating in outdoor activities [77].

#### Near vision tasks and myopia

Near work is traditionally considered as paper-based reading and writing at close distances. Nevertheless, the last few decades were marked by the adoption of digital devices in every aspect of human society, daily living, and activity. Several studies like the OLSM, SMS, SAVES, and others [46, 61, 177–179] explored the association of myopia with near work using parental surveys on activities such as school assignments, digital device use, and watching television. Myopic children were found to spend more time studying, reading, and writing compared to non-myopic children [46, 177]. A similar trend of additional near-work time was reported among urban children with a higher prevalence of myopia than rural children with lower myopia prevalence [177]. Children reading more than two books per week were also found to have three times higher risk of developing myopia than those reading less [179]. The intensity of near work [178], continuous reading (>30min), and closer working distance (<30cm) were also associated with an increased risk of myopia [61]. Meta-analysis of studies across five continents found near work to be associated with a greater risk (OR, 1.14) of myopia, with a significant more reading time (but not studying, watching television, or computer use) among myopes [180]. Yet, two recent meta-analyses found insufficient evidence of a definite risk between myopia and digital screen time [181, 182]. Even though the impact of digital device use (“screen time” estimated using a parental questionnaire) on childhood myopia failed to find any significant association between the two, an increase in myopic refraction by 0.28–0.33 D was observed for every hour spent in digital devices (smartphone and computer) [183].

Shifting away from the limitations of relying solely on questionnaires to evaluate near work, recent studies employing more objective measurement methods have revealed that myopic children tend to engage in near activities, specifically those closer than 20 cm, for extended durations, as determined using devices like Clouclip [184]. Additionally, these studies have indicated that myopic children also utilize double the smartphone data, indicating prolonged screen time, compared to their non-myopic counterparts [185]. Others have found that only 10 min or more of 2.5 D accommodative task at downward gaze was sufficient to stimulate axial elongation [186].

On the other hand, longitudinal studies [59, 62, 85, 187] found no association of near work or number of books read in children. Outdoor activity, or lack of it, was hypothesized to have a stronger influence on the development of myopia than near work itself and their combined effect may be a better biomarker of myopia. Studying the combined effect of time outdoors and near work on myopia, “SMS” reported a protective effect of high time outdoors in children performing high levels of near work [57]. The follow-up study “SAVES” reported that in young children, spending less time outdoors and engaging in high levels of near work at age 6 significantly increased the odds of developing myopia by age 12 (OR, 15.9). This risk remained elevated even for those with moderate (OR, 7.9) or low (OR, 5.3) levels of near work. In an older cohort, a similar trend was observed. Less outdoor time combined with high near work at age 12 conferred an increased risk of myopia by age 17 (OR, 5.1). However, only those who spent more time outdoors were protected, while the risk was not significantly altered by variations in near work (moderate: OR, 2.45; high: OR, 2.27). Conversely, spending moderate to low time outdoors at baseline significantly increased the myopia risk by over 3-fold, regardless of near-work levels [61].

#### Potential mechanisms of near work-related risk of myopia

##### Accommodation

As mentioned earlier in this section, a potential explanation to the effect of near work on myopia development is the changes in ocular accommodation status like lag (difference between accommodative demand and response) or fluctuation (standard deviation during sustained accommodation) [188]. Results from studies associating myopia and accommodative lag are mixed with reports of both lower amplitude compared to non-myopes [189, 190] and no association between the two [191]. Similarly, studies prescribing bifocals and progressive addition lenses (PALS) to reduce accommodative lag in myopes found mixed results with both clinically significant and non-significant reductions in myopia progression [192–194]. Moreover, these lenses also impose relative peripheral myopia alongside reducing the accommodative lag, and it is not clear whether this change in peripheral refraction or the accommodation influenced the result [195]. Likewise, accommodative micro-fluctuation is thought to increase in myopes because of an increase in aberration and reduction in blur sensitivity [196] and could generate hyperopic defocus and retinal blur, resulting in relative form-deprivation myopia. However, the current literature does not conclusively support this evidence [197, 198]. The understanding of how accommodation contributes to the development and progression of myopia remains elusive [195].

##### Relative peripheral refraction

Accommodation shifts the hyperopically defocused image (behind the retina) during near work and focusses on the retina by the forward movement of the ciliary body and lens shape alteration [199]. Although the accommodation system works in response to foveal image defocus (blur) during near work, the resultant refractive change transpires for the entire retina. While performing near tasks, although the foveal image is pulled forward and focused on the retina, the curved shape (prolate, flatter retina in the periphery) of the retina results in relative hyperopic defocus at the periphery which stimulates axial elongation and consequently myopia [200]. This relative peripheral hyperopic shift was demonstrated in several studies where the ocular shape became more prolate with accommodation and was hypothesized to be influenced by increased tension in the choroid [201]. Conversely, some studies found a relative peripheral myopic shift in myopes [202] while others found no shift in peripheral refraction in response to accommodative demand of up to 3 D [203]. In addition, pupil constriction associated with high-intensity of light outdoors and proximity to objects results in an increase in depth-of-focus and consequent reduction in peripheral defocus, aberration, and image blur [204–206]. Higher-order ocular aberration, especially chromatic and spherical aberrations, are also related to axial elongation in myopic eyes [207–209]. Overall, there is a lack of consistency in the results possibly due to differences in study design and cohort of choice.

### Urbanization and housing type

Myopia prevalence is associated with urban areas and high population density [59, 61, 67, 210–213]. In fact, countries known for their rapid urbanization, such as China, Singapore, and South Korea, have a high prevalence of myopia, ranging from 69 to 73% [7, 214, 215]. In addition, housing type, housing size, and living floor were revealed to influence myopia development [216, 217]. Myopia was more prevalent among children living (1) in apartments rather than in separate houses, (2) on higher floors compared to those living on lower floors, and (3) in large dwelling spaces [217–219]. However, housing types can be confounded by the level of education, income, and occupation [219], as living on higher floors and larger apartments in cities may contribute to the reduction of time spent outdoors and an increase in near work.

### Pollution

Air pollutants such as carbon monoxide, nitrogen oxides, and ozone are hypothesized to damage the ocular tissue, reducing the release of DA and causing systemic inflammation, oxidative stress, retinal ischemia, and resultant myopia [220]. Fine particulate matter (PM_2.5_) and ozone (O_3_) were also found to have an additive effect on myopia development, albeit in an elderly population [221]. The association between myopia and traffic-related air pollutants PM2.5 and nitrogen oxides was found in a study conducted among 15,822 Taiwanese [222] children and 2727 Brazilian [223] schoolchildren. However, it is important to note that this association is confounded by the fact that air pollution is often prevalent in urban areas, which are also characterized by less green spaces, low frequency visual environment, reduced time spent outdoors, disrupted sleep, all of which may contribute to myopia development.

### Second-hand smoking

Earlier studies with uncontrolled confounders like age, parental education, socio-economic status, and housing type presented conflicting associations between smoking and myopia [224–228]. The possible pharmacological causal pathway mediated via both nicotinic agonist and antagonist was also ill-defined [229, 230]. Recently, a large cross-sectional study on 6–8-year-old Hong Kong children has shown an association of second-hand smoking exposure with both the onset and progression of myopia [231]. A small but dose-response effect of smoking was observed with an increase in 0.07 D myopia or 0.04 mm AL elongation with 10 cigarettes/day [231]. The study was cross-sectional in design and could not prove a causal relationship.

### Seasons

It is unclear whether myopia onset and progression show seasonal variations, potentially due to variations in total light exposure [92]. The Correction of Myopia Evaluation Trial (COMET) [232] found that the rate of myopia progression and axial elongation decreased during summer and increased during winter, corresponding to individual light exposure levels [233]. Interestingly, the season of a child’s birth was also associated with his/her risk of developing myopia, with a clearer link especially in high myopes. Two large-scale studies in Israel (*n*=276,911, 16–22 years old) [234] and the UK (*n*=74,459, 18–100 years old) [235] observed a higher prevalence of myopia among participants born in summer/autumn than those born in winter. However, there was little association between myopia risk and photoperiod. Subsequent studies in China reported different findings, i.e., lower spherical equivalent or more myopic refraction in children exposed to the longest photoperiod by 0.44 D (*n*=722, 1–3 months old) [236]. Another study (*n*=1222, 0–3 years old) [237] reported 0.12 D more myopic refraction in children born in winter compared to children born in summer. This association has been hypothesized to involve factors like perinatal light exposure, melatonin production, birth weight, and temperature. Multiple confounding factors make it challenging to definitively establish the relationship between seasons and myopia.

### Lifestyle and parental factors

#### Physical activity

Physical activity (PA) measured using both objective (i.e., accelerometers) and subjective (i.e., questionnaires) means was shown not to be associated with myopia development [211, 238]. Others reported PA to be inversely correlated with myopic refractive change [239], whereas ALSPAC [60] found PA to decrease the risk of incident myopia when done outdoors, concluding that the reported PA is mainly capturing information related to time outdoors. Recent reviews further underscore the role of increased outdoor time, rather than PA itself, in controlling myopia progression [77, 240]. Nonetheless, promoting sports and physical activity is still beneficial for encouraging children to spend more time outdoors, given the protective effect of outdoor time in reducing myopia progression [211]. Furthermore, the use of the word “sports” in questionnaires instead of PA, and its misinterpretation as only physically demanding exercise or games led to the categorization of outdoor cycling and walking as leisure time activity [240]. The influence of PA is confounded by the fact that most PA is likely to occur outdoors and its protective effect against myopia is observed with more active outdoor PA [77, 240].

#### Sleep and circadian rhythms

Circadian rhythms are internal physiological and behavioral bodily processes that follow a roughly 24-h cycle [241]. These rhythms, generated by multiple oscillators in the body, are synchronized by the central biological clock located in the suprachiasmatic nucleus (SCN). The dominant cue for entrainment of the SCN, and consequently other bodily circadian rhythms in humans and other mammals, is the light/dark cycle (for review see Najjar and Zeitzer [241]). The SCN controls the rhythmicity of the pineal gland responsible for melatonin secretion through both photic and non-photic inputs [242, 243]. Thus, the melatonin secretion profile, more specifically, dim light melatonin onset (DLMO) can be a reliable endogenous biomarker of the circadian phase or circadian entrainment [244]. Even though DLMO has been linked with myopia, findings are conflicting, with evidence of both differences [245] and no differences [246] in the DLMO phase, along with variable salivary and urinary melatonin amplitudes between different refractive groups [247]. Recently, Chakraborty and colleagues elegantly reported that myopic children exhibit a significant DLMO phase delay (~1h) and lower aMT6s urinary melatonin levels compared to emmetropes [248]. Since melatonin levels are very sensitive to light [247], studies with robust methodological designs under controlled lighting conditions are essential to establish any relationship between melatonin dysregulation and myopia development. It is also worth mentioning that experimental work has also shown that the absence of circadian time cues (e.g., constant light or constant darkness) can disrupt ocular circadian rhythms. In young rapidly growing eyes, this disruption often results in aberrant eye growth and failure to achieve emmetropization [249].

Sleep is under circadian and homoeostatic control and may also contribute to ocular growth and emmetropization [250]. Myopic children have recently been reported to exhibit delays in sleep onset and wake-up time, which aligns with delays in DLMO [248], in addition to reduced sleep quality [248, 251] compared to emmetropes. Furthermore, it has been postulated that lack of sleep or later bedtime could lead to additional near work, and thus higher risk for myopia [252]. To date, however, associations between sleep disorders (e.g., insufficient duration, poor quality, irregular, and late timing of sleep) and the incidence and progression of myopia remain deficient [253]. This is because most studies were limited by insensitive outcome measures, differences in the definition of studied variables and participant demographics [253], the lack of cycloplegic refraction leading to overestimation of myopia, and recall bias from questionnaires estimating sleep characteristics [254].

In summary, the current body of evidence seeking to establish associations between sleep, circadian rhythms, and myopia still demonstrates a lack of robustness. To strengthen the validity of these findings, it is imperative to conduct further longitudinal studies that adhere to universally accepted definitions of sleep quality and myopia. Additionally, the incorporation of objective measures for assessing sleep, light exposure, and near work is crucial for accurately confirming any associations between sleep, circadian rhythms, and myopia [252, 254].

#### Diet and nutrition

The relationship between diet and myopia is controversial. Whole grain, higher saturated fat, refined carbohydrates, and cholesterol intake were linked with greater axial growth and myopia [255–257]. In contrast, other studies found no association between the development of childhood myopia with vitamin A, protein, fat, and carbohydrate in diets [258–260]. As suggested earlier in the “vitamin D” sub-section under the “Potential mechanisms for light-induced myopia control”, vitamin D probably offers no protection towards myopia and its blood serum level only indicates sunlight exposure [166, 167].

In a retrospective analysis of 6855 individuals aged 12 to 25 years, no significant association with myopia was found for nutritional factors like serum vitamin D, glucose levels, or caffeine intake, except for increased insulin levels, which were related to a higher likelihood of having myopia [261]. In addition, a systematic review revealed that most studies on nutrients and dietary associations with myopia are non-interventional and provide inconsistent evidence of a connection [262]. Given the complexity of diet and nutrition, more structured investigations are necessary to fully comprehend any potential associations with myopia.

#### Socioeconomic status and level of education

Socioeconomic status is defined by several factors such as parental education, employment, income, accessibility of services, school fees (private vs government), and housing type [187, 263–265]. A large-scale study in China found a positive correlation between myopia and higher socioeconomic status indicators such as urban living, owning property, and duration of education [266]. The authors proposed that economic development fosters a desire for wealth, leading to increased educational pursuit and heavier academic burdens, ultimately resulting in higher myopia rates [266]. While other studies found higher socioeconomic status to be associated with myopia [264, 265, 267, 268], some failed to find any such association [61, 187, 269]. The conflicting evidence on socioeconomic status and myopia may stem from variations in the definition and classification of socioeconomic status, as well as unmeasurable factors like parental involvement and academic pressure. More importantly, the effect of socioeconomic status can be due to more near time and less time outdoors.

As previously discussed in the “Near visual task and myopia” section, intensive near work, its duration, and close working distances have been linked to an increased risk of myopia and AL elongation. Consequently, the level of education (measured as years of education) is also a risk factor for myopia [270–272]. Even higher intelligence quotient and better school performance were observed to be positively associated with myopia [273–275]. Recent studies suggested that not only does the child’s educational level affect their ocular development and growth, but parental educational levels can also be a significant risk factor for the development of myopia in children [276]. However, it is important to note that this relationship is confounded by factors like parental myopia (genetics), income, and occupation, reduced time outdoors [277].

Myopia is a multifactorial and complex condition affected by several environmental factors in isolation or combination. The summary of evidence from the literature on the environmental factors influencing myopia, the quality of evidence, and their relationship with myopia documented so far is listed in Table 1.

**Table 1:** Summary of environmental and lifestyle factors influencing myopia

Factor	Evidence	Relationship with myopia
Time spent outdoors	Strong	• Increasing time outdoors is associated with less risk of myopia onset. • Dose-dependent effect.
Light intensity or light levels	Strong	• Higher levels of light are associated with less myopia. • Potentially a dose-dependent effect.
Spectral composition of light	Possible	• Interventions using red, blue, or violet lights have shown promising results. • These findings require further evaluation with a longer study duration, better side-effect evaluation, and possible rebound effect investigation.
Timing, duration, pattern of light exposure	Possible	• Protective, duration-dependent effect of high illuminance light. • The impacts of timing and patterns of light exposure are understudied in humans and limited to animal studies.
Spatial frequency of the visual environment	Possible	• Lower spatial frequency is associated with an increased risk of myopia development. • Findings are limited to animal studies and mathematical modeling in humans.
Physical activity	Weak	• Not an independent factor but rather linked to time spent outdoors.
Near work	Strong	• Intensity, continuity, and closer working distance are consistently associated with a higher risk of myopia.
Accommodation	Possible	• Inconsistent evidence on accommodation lag and amplitude. • Impacts are not fully understood.
Relative peripheral refraction	Possible	• Peripheral retinal hyperopic defocus is associated with myopia development. • These findings lack consistency.
Urbanization and housing	Weak	• Inconsistent and related to increased near work and reduced time spent outdoors.
Socioeconomic status	Weak	• Inconsistent and related to increased near work.
Education level	Possible	• Predominantly related to increased near work.
Pollution	Weak	• Related to urbanization and housing type and increased near work.
Second-hand smoking	Weak	• Inconsistent and weak association between smoking and myopia. • Confounded by education and subjective measurements.
Seasons	Weak	• Children born in summer have a lower incidence of myopia. • Less myopia progression was observed during summer. • Unclear link, confounded by multiple factors.
Sleep and circadian rhythms	Possible	• Potential link between delayed circadian phase and myopia. • Inconsistent and conflicting evidence which needs objective quantification of sleep and light exposure.
Diet and nutrition	Weak	• Inconsistent and needs more structured investigations.

## Current treatment options for myopia control

While the refinement of environmental features and lifestyle remains the best approach for preventing or delaying the onset of myopia, today, a variety of optical and pharmacological treatment options are available to slow the progression of myopia. These include optical interventions using multifocal contact lenses, myopia control spectacles using defocus incorporated multiple segments, orthokeratology, and pharmaceutical intervention using a low-dose atropine [278, 279].

## Conclusions

While genetics certainly play a role in the development of myopia, it is crucial to recognize the substantial impact of both the visual and non-visual environments in shaping its progression. The time spent outdoors and engagement in near-work activities stand out as the most influential, independent, determinants of myopia, with contrasting effects—the former protective, the latter exacerbating the condition. However, our understanding of the intricate relationship between various light attributes, visual environments, and myopia remains limited—further studies need to be undertaken. Similarly, the association between lifestyle factors like sleep and nutrition and myopia remains a topic of debate, also warranting further investigation. To effectively address childhood myopia, we must understand the complex interplay between outdoor activities, near visual tasks, and other environmental and lifestyle factors. This holistic approach will enable the development of tailored protective strategies including the refinement of the indoor environment (lighting, spatial frequency, etc.) for myopia prevention.

## Data Availability

Data will be made available upon reasonable request to the corresponding author.

## Abbreviations

AL: Axial length
D: Diopter
OCT: Optical coherence tomography
RPE: Retinal pigment epithelium
EDI: Enhanced depth imaging
FDM: Form deprivation myopia
GWAS: Genome-wide association studies
SNP: Single nucleotide polymorphism
SMS: Sydney myopia study
SCORM: Singapore Cohort of Risk factors for Myopia
BMPS: Beijing Myopia Progression Study
ALSPAC: Avon Longitudinal Study of Parents and Children
SAVES: Sydney Adolescent Vascular and Eye Study
CLEERE: Collaborative Longitudinal Evaluation of Ethnicity and Race
OLSM: Orinda Longitudinal Study of Myopia
RCT: Randomized controlled trial
ACES: Anyang Childhood Eye Study
COVID: Coronavirus disease
CT: Choroidal thickness
LED: Light-emitting diodes
UV: Ultraviolet
IR: Infrared
DA: Dopamine
HT: Hydroxytryptamine
GABA: Gamma-aminobutyric acid
RA: Retinoic acid
ipRGC: Intrinsically photosensitive retinal ganglion cell
PALS: Progressive addition lenses
COMET: Correction of Myopia Evaluation Trial
PM: Particulate matter
O_3_: Ozone
PA: Physical activity
SCN: Suprachiasmatic nucleus
DIMS: Defocus incorporated multiple segments
ATOM: Atropine for the Treatment of Childhood Myopia
LAMP: Low concentration atropine for myopia progression
SCN: Suprachiasmatic nucleus
DLMO: Dim-light melatonin onset
